# Utilization, user evaluation, and associated factors of Traditional Chinese Medicine techniques for insomnia symptoms among Chinese community-dwelling older adults: a cross-sectional study

**DOI:** 10.3389/fpubh.2025.1746822

**Published:** 2026-01-15

**Authors:** Yun Mo, Li Hu, Xi Shen, Yi Zhang, Site Li, Jiaxiu Peng, Yuping Long, Xiangrong Wu, Binbin Xu

**Affiliations:** 1 School of Nursing, Hunan University of Chinese Medicine, Changsha, Hunan, China; 2 The Affiliated Hospital of Guizhou Medical University, Guiyang, Guizhou, China

**Keywords:** Andersen Behavioral Model, community, healthcare utilization, insomnia symptoms, older adults, Traditional Chinese Medicine (TCM)

## Abstract

**Background:**

Insomnia symptoms are common among older adults, and Traditional Chinese Medicine (TCM) techniques have shown benefits in relieving these symptoms. However, real-world evidence on their use, evaluation, and associated factors in community settings remains limited. This study aimed to describe TCM use and user evaluation for insomnia symptoms among community-dwelling older adults and to explore associated factors.

**Methods:**

An exploratory cross-sectional survey was conducted from November 2024 to April 2025 in 15 communities in Changsha, China. Older adults (≥60 years) with clinically significant insomnia symptoms (Insomnia Severity Index ≥8) were recruited. Data were collected on TCM use, perceived satisfaction and effectiveness, and predisposing, enabling, and need-related factors based on the Andersen Behavioral Model. Logistic regression was used to identify associated factors.

**Results:**

Among 311 participants, 51.1% reported using at least one TCM technique, most commonly tuina (71.1%) and moxibustion (69.8%). Satisfaction was generally high, though only 20.1–28.3% were “very satisfied” and 22.6% perceived the interventions as “highly effective.” Four factors were significantly associated with TCM use: pre-retirement occupation and awareness of TCM techniques (predisposing), having relatives or friends engaged in TCM-related industries (enabling), and insomnia severity (need).

**Conclusion:**

This study provides preliminary evidence on the real-world use of TCM techniques for insomnia symptoms among community- dwelling older adults. More than half of participants reported using at least one TCM technique, yet only a minority rated the TCM services as “very satisfied” or “highly effective.” Pre-retirement occupation, awareness of TCM techniques, having relatives or friends working in TCM-related industries, and insomnia severity were associated with TCM technique use. These findings may inform the design of culturally sensitive interventions to manage insomnia symptoms among community-dwelling older adults in similar contexts. Future research should further examine implementation quality and ways to improve both utilization and user satisfaction.

## 1 Introduction

Insomnia disorder is the most common sleep–wake disorder, defined by persistent difficulty initiating or maintaining sleep or experiencing early morning awakenings despite adequate opportunity for sleep, accompanied by clinically significant daytime impairment (1, 2). Diagnostic guidelines specify that these symptoms must occur at least three nights per week and persist for at least 3 months to confirm a diagnosis (1, 3). Its prevalence increases with age (1), posing a substantial public health challenge. Among adults aged ≥60 years, the prevalence of clinically diagnosed insomnia disorder ranges from 12 to 20% (2), compared to 6–10% in the general population (1). This elevated risk is closely associated with age-related physiological changes, multimorbidity, decreased mobility, retirement, and reduced social interaction (2).

The consequences of insomnia disorder extend far beyond nighttime sleep disturbance, affecting multiple health domains. Evidence indicates that insomnia disorder is a significant risk factor for hypertension, diabetes, obesity, and cardiovascular disease (4–6), as well as mood disorders such as depression and anxiety (7, 8). A meta-analysis links it to an increased risk of cognitive decline and dementia (9), while other studies associate it with falls (10, 11), frailty (12), reduced quality of life (13), and increased healthcare utilization and costs (14). Addressing insomnia in older adults is therefore essential to promote healthy aging and reduce societal disease burden.

Compared with insomnia disorder, insomnia symptoms—frequent difficulty falling or staying asleep without meeting full diagnostic criteria—are even more widespread. Globally, these symptoms affect 30–48% of individuals aged ≥60 years (2). In China, prevalence varies by measurement tool and setting. For example, a study showed that 33.9% of Chinese community-dwelling adults aged≥60 years had insomnia symptoms, measured by the Pittsburgh Sleep Quality Index (12). The prevalence of insomnia symptoms was 45.7% among rural Chinese older adults aged 60 years and above, measured by the Insomnia Severity Index (ISI) (15). These symptoms may persist for months or years, substantially increasing health risks even without a formal diagnosis. For instance, a longitudinal cohort study examined the trajectories of insomnia symptoms over 12 months during a time of stress and uncertainty and found that insomnia symptoms demonstrated a high persistence rate of 54% over time; Only 10.05% of participants with insomnia symptoms at baseline remitted over 12 months (16). These findings underscore the critical need for timely management of insomnia symptoms, not just insomnia disorder.

Currently, cognitive behavioral therapy for insomnia (CBT-I) is the first-line treatment for insomnia across all age groups, while pharmacological therapies remain widely used (17, 18). However, both approaches face major challenges in older adults. Although CBT-I is the gold standard, its clinical uptake is very low, ranging from 1 to 29% (19). In China, its promotion is hindered by limited accessibility and acceptability: a severe shortage of qualified therapists (especially in primary care), cultural stigma toward psychological treatment, high costs, geographic disparities, and an underdeveloped mental health service system (20, 21). Current hypnotics (Z-drugs, benzodiazepines, low-dose doxepin, suvorexant) carry notable risks in older adults; thus, short-term or intermittent use is advised (22). Ramelteon may offer safer sleep-onset benefits but has limited efficacy for sleep maintenance, while melatonin is relatively safe but lacks robust safety data in older populations (22, 23). Additionally, many older adults perceive insomnia as a “normal” part of aging and avoid professional care; only 2.1% of adults with insomnia symptoms sought sleep-related healthcare in the past year (24). These structural and perceptual barriers highlight an urgent need for alternative, culturally acceptable, and scalable solutions, particularly in community settings.

Traditional Chinese Medicine (TCM) techniques, grounded in the principles of yin-yang balance, five elements, and holistic regulation, offer promising options for managing insomnia symptoms, especially among older adults in community settings (25, 26). Common techniques—such as acupuncture, tuina, moxibustion, auricular acupressure, and needle embedding—are generally safe, low-cost, easy to administer, and culturally appropriate (27). Randomized controlled trials and systematic reviews have provided preliminary evidence supporting their clinical effectiveness in improving sleep quality (25–30). However, existing research has largely focused on efficacy under controlled conditions, with little attention to real-world implementation. In particular, little is known about how older adults in community settings actually use different TCM techniques, how they perceive the effectiveness of TCM techniques and their service experience, and what factors affect their use. Moreover, previous studies have rarely applied theoretical frameworks to systematically explain TCM utilization behaviors, limiting the interpretability and transferability of findings across settings.

To address these gaps, this study aimed to (1) describe the patterns of TCM technique use and users’ perceived satisfaction and effectiveness among older adults with insomnia symptoms, and (2) explore the associations between TCM technique use and a range of factors, as conceptualized by the Andersen Behavioral Model (ABM) —a widely applied framework for explaining healthcare utilization based on predisposing, enabling, and need factors (31). Predisposing factors reflect an individual’s tendency to use healthcare services and include demographics, social structure, and health beliefs; Enabling factors refer to resources and conditions that facilitate or hinder access, such as personal, family, and community resources; Need factors represent the immediate reasons for service use, including perceived and actual health status (31). In this study, 13 potential associated factors were examined: eight predisposing factors (sex, age, marital status, education level, pre-retirement occupation, place of residence, awareness of TCM techniques, and attitude toward TCM techniques), four enabling factors (monthly personal income, type of medical insurance, availability of TCM medical institutions within 5 km, and having relatives or friends working in TCM-related industries), and one need factor (insomnia severity).

By addressing these gaps, this study contributes to existing literature in several ways. First, it provides real-world evidence on utilization and user evaluation of TCM techniques for insomnia symptoms in community settings, where most older adults actually seek care. Second, by applying the ABM, this study offers a theoretically grounded framework for understanding TCM utilization behaviors, thereby facilitating interpretation of findings and enhancing their relevance to other community and primary care contexts. Third, by focusing on older adults with insomnia symptoms rather than clinically diagnosed insomnia, the study captures an often overlooked but high-risk population, offering insights that may inform the design of scalable, culturally appropriate, community-based interventions to support healthy aging.

## 2 Materials and methods

### 2.1 Study design and setting

We conducted a community-based cross-sectional study in 15 communities in Changsha, Hunan province, China from November 2024 to April 2025.

### 2.2 Participants

Participants were eligible if they were aged 60 years or older, had insomnia symptoms as assessed by the ISI (score ≥8 indicating clinically significant insomnia symptoms) (32–35), and were able to communicate in Mandarin. Exclusion criteria were: (1) insomnia secondary to alcohol use, substance abuse or dependence, or medication side effects; (2) insomnia caused by acute environmental changes (e.g., relocation, jet lag, noise); or (3) shift workers or individuals with long-term night shift work.

### 2.3 Sample size and sampling methods

The required sample size was calculated using the single-sample rate estimation formula:


$$n={\left(\frac{z_{1-\frac{\alpha }{2}}}{\delta }\right)}^{2}\cdot p\cdot (1-p)$$

Where p represents the expected prevalence of TCM technique use, *δ* denotes the allowable margin of error (typically set at 3–5%), and *α* is the probability of a Type I error, set at 0.05 (two-tailed). Based on a previous study reporting a prevalence of 17.7% among older adults aged 60 years and above (36), and with *α* = 0.05 and *δ* = 5%, the calculated minimum sample size was 224. Allowing an anticipated non-response rate of 20%, a minimum of 280 (=224/0.8) participants was required. In addition, to ensure the stability of the binary logistic regression analysis, we assessed whether the final sample size satisfied the commonly recommended criterion of at least 10 outcome events per estimated parameter (37). The available number of outcome events was sufficient to support the planned regression analyses.

A convenience sampling method was used to recruit the participants. To enhance the diversity of the sample, recruitment was conducted across 15 geographically dispersed communities in Changsha, encompassing both urban and rural neighborhoods. Within these communities, researchers approached potential participants at various public spaces, including community activity centers, parks, and residences, on different days of the week and times of the day.

### 2.4 Variables and measures

The full version of the questionnaires used in this study is provided in Supplementary material.

#### 2.4.1 Dependent variable

The dependent variable was the use of TCM techniques, assessed by the question: “Have you ever used TCM techniques to relieve insomnia symptoms in the past 12 months? (Yes/No).” Participants who answered “Yes” were additionally asked about the type, frequency, and location of TCM technique use, as well as their satisfaction across seven domains: overall service, professionalism, cost, convenience, safety, privacy protection, and effectiveness in relieving insomnia symptoms.

#### 2.4.2 Independent variables

General characteristics were collected through a self-designed questionnaire: including sex, age, marital status, education level, occupation before retirement, monthly personal income, type of medical insurance, place of residence, availability of TCM medical institutions within 5 km of the residence, and having relatives or friends engaged in TCM-related industries.

Awareness of TCM techniques was assessed using a six-item questionnaire developed by the research team based on literature review and expert discussion (38–40). The items covered knowledge of TCM technique types, mechanisms, procedures, safety, application for insomnia relief, and relevant policies. Each item was rated on a 4-point Likert scale (1 = Completely unaware to 4 = Very aware), yielding a total score ranging from 6 to 24, with higher scores indicating greater awareness. Awareness levels were categorized as low (≤25th percentile), medium (26th–75th percentile), and high (≥76th percentile). The scale demonstrated good internal consistency in this study (Cronbach’s *α* = 0.810) and a satisfactory scale content validity index of 0.861. Attitude toward TCM techniques was assessed by one item: “Overall, what is your attitude toward TCM techniques? (Very opposed/Not very supportive/Relatively supportive/Very supportive).”

Insomnia severity was measured using the ISI (32–35), a widely used 7-item instrument assessing insomnia severity, satisfaction with sleep, functional impact, awareness of consequences, and related distress. Each item is scored on a 0–4 scale, yielding a total score of 0–28, with higher scores indicating more severe insomnia (0–7: no clinical insomnia; 8–14: mild; 15–21: moderate; 22–28: severe). The Chinese version of ISI has demonstrated good reliability and validity, with Cronbach’s *α* = 0.81 (41).

### 2.5 Data collection procedures

Data was collected by five third-year undergraduate students who had completed the Nursing Research course, received standardized training, and had prior experience in data collection. Following ethical approval, on-site data collection was carried out at community activity centers, parks, and residences in the 15 selected communities using paper-based questionnaires.

Potential participants were screened for eligibility, and the study objectives and procedures were explained in detail. Written informed consent was obtained from all participants prior to recruitment. During data collection, trained interviewers provided one-on-one assistance to ensure accurate and complete responses. Upon completion, questionnaires were immediately reviewed for completeness, and any missing or unclear responses were clarified with participants on the spot. Each participant received a small gift (a pack of tissues) as a token of appreciation for their time and participation.

### 2.6 Data analysis

Data analysis was conducted using IBM SPSS 25.0 (IBM Corp., Armonk, NY, USA). Participants’ characteristics and the use and satisfaction of TCM techniques were presented as frequency and percentage. Backward stepwise binary logistic regression was used to identify factors associated with the use of TCM techniques, with *p* > 0.05 as the removal criterion. 𝝌2 tests were used to screen candidate influencing factors, and variables with *p* < 0.25 in 𝝌2 tests were included in the regression model (42). Prior to conducting binary logistic regression, we assessed multicollinearity among candidate independent variables by calculating the variance inflation factor (VIF). A VIF value exceeding 5 was considered indicative of moderate multicollinearity, and exceeding 10 indicated severe multicollinearity. In our data, all VIF values were below 5, suggesting no significant multicollinearity that would compromise the stability of the regression estimates (Supplementary Table S1). Statistical significance was set at 0.05 (two-sided).

## 3 Results

### 3.1 Participants’ characteristics

We approached 1,167 older adults from the community, of whom 342 met the eligibility criteria. Ultimately, 311 agreed to participate and completed the survey (response rate: 90.9%).

Slightly more than half were female (59.2%), and 71.4% were aged 65 years or older. About two-thirds (66.9%) were married, whereas only 10.3% had attained an education level of junior college or above. The largest occupational category before retirement was enterprise employees/workers (39.2%), followed by farmers (33.1%). Most participants (80.8%) reported a monthly personal income below 4,000 Chinese yuan, and over half (53.1%) were covered by Urban–Rural Resident Basic Medical Insurance. A majority lived in urban areas (62.1%), nearly half (48.2%) reported access to TCM medical institutions within 5 km of their residence, and 27.7% had relatives or friends engaged in TCM-related industries. Regarding knowledge and attitudes, 64.3% demonstrated medium to high awareness of TCM techniques, and 84.9% expressed supportive attitudes toward their use. In terms of sleep health, 87.2% reported moderate to severe insomnia (Table 1).

**Table 1:** Participants characteristics (*N* = 311).

Characteristic	*n* (%)
Sex	
Male	127 (40.8)
Female	184 (59.2)
Age (years)	
60–64	89 (28.6)
65–69	87 (28.0)
70–74	73 (23.5)
≥75	62 (19.9)
Marital status	
Married	208 (66.9)
Unmarried/Divorced/Widowed	103 (33.1)
Education level	
Primary school or below	123 (39.5)
Junior high school	84 (27.0)
High school/Technical secondary/Vocational school	72 (23.2)
Junior college or above	32 (10.3)
Occupation before retirement	
Civil servants/Public institution staff	61 (19.6)
Enterprise employees/Workers	122 (39.2)
Self-employed	25 (8.0)
Farmers	103 (33.1)
Monthly personal income (Chinese yuan)	
<2000	110 (35.4)
2000–2,999	72 (23.2)
3,000–3,999	69 (22.2)
≥4,000	60 (19.3)
Type of medical insurance	
Urban Employee Basic Medical Insurance	130 (41.8)
Urban–Rural Resident Basic Medical Insurance	165 (53.1)
No insurance	16 (5.1)
Place of residence	
Urban areas	193 (62.1)
Rural areas	118 (37.9)
Presence of TCM medical institutions within 5 km of the residence^*^	
Yes	150 (48.2)
No	96 (30.9)
Not sure	65 (20.9)
Relatives/friends working in TCM-related industries	
Yes	86 (27.7)
No	225 (72.3)
Awareness of TCM techniques	
Low	111 (35.7)
Medium	135 (43.4)
High	65 (20.9)
Attitude toward TCM techniques	
Very opposed / Not very supportive	47 (15.1)
Relatively supportive/Very supportive	264 (84.9)
Insomnia severity	
Mild	40 (12.9)
Moderate	171 (55.0)
Severe	100 (32.2)

TCM, Traditional Chinese Medicine.

^*^Includes TCM hospitals, TCM departments of general hospitals, and integrated traditional Chinese and Western medicine hospitals.

### 3.2 Use and evaluation of TCM techniques

Among 311 participants, 51.1% reported using TCM techniques for insomnia. The most commonly used approaches were tuina (71.1%), moxibustion (69.8%), needle embedding (59.1%), and acupuncture (58.5%), followed by auricular acupressure (39.6%). Regarding utilization frequency, 34.6% used the techniques occasionally and 29.6% used them 1–2 times per month. Most participants sought services at TCM hospitals (72.3%), private clinics (50.3%), or general hospital TCM departments (47.2%), while around one-third reported accessing services at wellness centers, community health service centers, or at home. Regarding satisfaction (overall service, professionalism, cost, convenience, safety, privacy protection, and insomnia relief effectiveness), 69.8–81.8% of participants expressed satisfaction. However, only 20.1–28.3% rated themselves as “very satisfied” across these dimensions, indicating that high overall satisfaction coexisted with relatively low proportions of high-level satisfaction (Table 2).

**Table 2:** Use and satisfaction of TCM techniques among older adults with insomnia (*N* = 311).

Variables	*n* (%)
Use experience	
No	152 (48.9)
Yes	159 (51.1)
Type of techniques used^*^	
Tuina (therapeutic massage)	113 (71.1)
Moxibustion	111 (69.8)
Needle embedding	94 (59.1)
Acupuncture	93 (58.5)
Auricular acupressure (ear seeds)	63 (39.6)
Others	60 (37.8)
Frequency of use^#^	
Daily	20 (12.6)
1–2 times per week	37 (23.3)
1–2 times per month	47 (29.6)
Occasionally (≤10 times/year)	55 (34.6)
Places of service^*^	
TCM hospital	115 (72.3)
Private TCM clinic	80 (50.3)
TCM department of general hospital	75 (47.2)
Wellness center	63 (39.6)
Community health service center	62 (39.0)
At home	55 (34.6)
Overall satisfaction with services^#^	
Very satisfied	32 (20.1)
Satisfied	87 (54.7)
Dissatisfied	30 (18.9)
Very dissatisfied	10 (6.3)
Professionalism rating^#^	
Very satisfied	45 (28.3)
Satisfied	76 (47.8)
Dissatisfied	27 (17.0)
Very dissatisfied	11 (6.9)
Cost satisfaction^#^	
Very satisfied	35 (22.0)
Satisfied	76 (47.8)
Dissatisfied	30 (18.9)
Very dissatisfied	18 (11.3)
Convenience rating^#^	
Very satisfied	38 (23.9)
Satisfied	76 (47.8)
Dissatisfied	35 (22.0)
Very dissatisfied	10 (6.3)
Safety rating	
Very satisfied	40 (25.2)
Satisfied	90 (56.6)
Dissatisfied	20 (12.6)
Very dissatisfied	9 (5.7)
Privacy protection satisfaction^#^	
Very satisfied	40 (25.2)
Satisfied	86 (54.1)
Dissatisfied	22 (13.8)
Very dissatisfied	11 (6.9)
Perceived relief of insomnia symptoms^#^	
Very satisfied	36 (22.6)
Satisfied	88 (55.3)
Dissatisfied	22 (13.8)
Very dissatisfied	13 (8.2)

TCM, Traditional Chinese Medicine.

^*^Multiple responses; percentages are based on actual users (*n* = 159), and totals can exceed 100%.

^#^Percentages are based on actual users (*n* = 159).

### 3.3 Associated factors of TCM technique use

Candidate variables identified in univariate analyses (Table 3, *p* < 0.25) were entered into a backward stepwise logistic regression model. Four independent associated factors of TCM technique use were identified (Table 4). Three variables entered into the initial model-education level, type of medical insurance, and place of residence-were not retained in the final model due to lack of independent statistical significance (see Supplementary Table S2 for details).

**Table 3:** Univariate analysis of factors associated with the use of TCM techniques (*N* = 311).

Factors	TCM techniques use		*χ^2^*	*p*
	No	Yes		
	*n* (%)	*n* (%)		
Sex			0.061	0.805
Male	61 (48.0)	66 (52.0)		
Female	91 (49.5)	93 (50.5)		
Age (years)			3.778	0.286
60–64	45 (50.6)	44 (49.4)		
65–69	35 (40.2)	52 (59.8)		
70–74	39 (53.4)	34 (46.6)		
≥75	33 (53.2)	29 (46.8)		
Marital status			0.104	0.747
Married	103 (49.5)	105 (50.5)		
Unmarried/Divorced/Widowed	49 (47.6)	54 (52.4)		
*Education level			10.085	0.018
Primary school or below	65 (52.8)	58 (47.2)		
Junior high school	48 (57.1)	36 (42.9)		
High school/Technical secondary/Vocational school	30 (41.7)	42 (58.3)		
Junior college or above	9 (28.1)	23 (71.9)		
*Occupation before retirement			25.359	<0.001
Civil servants/Public institution staff	25 (41.0)	36 (59.0)		
Enterprise employees/Workers	45 (36.9)	77 (63.1)		
Self-employed	11 (44.0)	14 (56.0)		
Farmers	71 (68.9)	32 (31.1)		
Monthly personal income (Chinese yuan)			3.300	0.348
<2000	57 (51.8)	53 (48.2)		
2000–2,999	30 (41.7)	42 (58.3)		
3,000–3,999	38 (55.1)	31 (44.9)		
≥4,000	27 (45.0)	33 (55.0)		
*Type of medical insurance			6.028	0.049
Urban Employee Basic Medical Insurance	53 (40.8)	77 (59.2)		
Urban–Rural Resident Basic Medical Insurance	91 (55.2)	74 (44.8)		
No insurance	8 (50.0)	8 (50.0)		
*Place of residence			8.306	0.004
Urban areas	82 (42.5)	111 (57.5)		
Rural areas	70 (59.3)	48 (40.7)		
Presence of TCM medical institutions within 5 km of the residence^#^			0.648	0.723
Yes	70 (46.7)	80 (53.3)		
No	48 (50.0)	48 (50.0)		
Not sure	34 (52.3)	31 (47.7)		
*Relatives/friends working in TCM-related industries			7.829	0.005
Yes	31 (36.0)	55 (64.0)		
No	121 (53.8)	104 (46.2)		
*Awareness of TCM techniques			81.893	<0.001
Low	89 (80.2)	22 (19.8)		
Medium	55 (40.7)	80 (59.3)		
High	8 (12.3)	57 (87.7)		
Attitude toward TCM techniques			0.095	0.758
Very opposed/Not very supportive	22 (46.8)	25 (53.2)		
Relatively supportive/Very supportive	130 (49.2)	134 (50.8)		
*Insomnia severity			28.813	<0.001
Mild	30 (75.0)	10 (25.0)		
Moderate	93 (54.4)	78 (45.6)		
Severe	29 (29.0)	71 (71.0)		

TCM, Traditional Chinese Medicine.

^*^Candidate-independent variable for multivariate analysis (*p* < 0.25).

^#^Includes TCM hospitals, TCM departments of general hospitals, and integrated traditional Chinese and Western medicine hospitals.

**Table 4:** Backward stepwise binary logistic regression of factors associated with the use of TCM techniques (*N* = 311).

Variables	B	SE.	*p*	Exp (B)	95% CI for EXP (B)	
					Lower	Upper
Predisposing factor						
Occupation before retirement						
Farmers	Reference					
Civil servants/Public institution staff	0.673	0.413	0.103	1.960	0.873	4.401
Enterprise employees/Workers	1.346	0.347	<0.001	3.843	1.948	7.580
Self-employed	0.488	0.552	0.377	1.629	0.552	4.810
Awareness of TCM techniques						
Low	Reference					
Medium	1.623	0.317	<0.001	5.069	2.723	9.434
High	3.229	0.480	<0.001	25.255	9.864	64.660
Enabling factor						
Relatives/friends working in TCM-related industries						
No	Reference					
Yes	0.847	0.327	0.010	2.332	1.228	4.428
Need factor						
Insomnia severity						
Mild	Reference					
Moderate	1.309	0.498	0.009	3.702	1.395	9.825
Severe	1.977	0.524	<0.001	7.224	2.586	20.178

TCM, Traditional Chinese Medicine; B, unstandardized regression coefficient; SE, standard error; Exp(B), odds ratio; CI, confidence interval.

Model summary: Cox & Snell R^2^ = 0.336; Nagelkerke R^2^ = 0.448; Omnibus test: Chi-square = 127.210, *p* < 0.001; Hosmer–Lemeshow test: Chi-square = 10.306, *p* = 0.172.

Among predisposing factors, occupation before retirement and TCM techniques awareness were significant. Compared with farmers, enterprise employees/workers showed higher odds of use (odds ratio [OR] = 3.84, 95% confidence interval [CI]: 1.95–7.58, *p* < 0.001). Those with medium awareness had fivefold higher odds (OR = 5.07, 95% CI: 2.72–9.43, *p* < 0.001), and those with high awareness had 25 times higher odds (OR = 25.26, 95% CI: 9.86–64.66, *p* < 0.001), compared with those with low awareness.

One enabling factor and one need factor were significant. Participants with relatives or friends engaged in TCM-related industries had greater odds of use compared with those without (OR = 2.33, 95% CI: 1.23–4.43, *p* = 0.01). Participants with moderate (OR = 3.70, 95% CI: 1.40–9.83, *p* = 0.009) and severe insomnia (OR = 7.22, 95% CI: 2.59–20.18, *p* < 0.001) were more likely to use TCM techniques than those with mild symptoms.

## 4 Discussion

This exploratory study provides initial real-world evidence on the use of TCM techniques for managing insomnia symptoms among community-dwelling older adults in China. We found that 51.1% of participants reported using at least one TCM technique, with tuina, moxibustion, needle embedding, and acupuncture being the most frequently used methods. Overall satisfaction with TCM services was generally high across domains such as safety and professionalism, yet fewer participants reported being “very satisfied.” Four associated factors of TCM use were identified based on the ABM: pre-retirement occupation and awareness of TCM techniques (predisposing); having relatives or friends working in TCM-related industries (enabling), and insomnia severity (need).

The prevalence of TCM technique use among older adults with insomnia symptoms in this study (51.1%) was substantially higher than the 17.7% reported in a previous study on TCM preventive service use among older adults (36). This discrepancy may be primarily attributed to differences in health needs: prior research focused on preventive care among individuals without acute health concerns, whereas our study targeted those with existing insomnia symptoms, likely increasing perceived need and motivation for service utilization. Additionally, participants were recruited from community activity centers, parks, or residences, which may underrepresent individuals who are less socially active or have more severe mobility limitations. Therefore, the observed usage rate of 51.1% should be interpreted with caution and needs verification in future studies employing probability sampling designs. When compared with the reported clinical uptake of CBT-I (1–29%) (19), TCM techniques use was significantly higher, underscoring their cultural acceptability and potential as community-based strategies for managing insomnia symptoms.

Regarding specific techniques, tuina (71.1%) and moxibustion (69.8%) were most frequently used, followed by needle embedding (59.1%) and acupuncture (58.5%), while auricular acupressure had the lowest uptake (39.6%). Notably, despite strong evidence supporting acupuncture and auricular acupressure for insomnia symptoms (27, 29), their real-world utilization was relatively low. Possible reasons include the minimally invasive nature of acupuncture, which may cause pain or fear of adverse events, and practical limitations of auricular acupressure such as discomfort, adhesive instability, and unfamiliarity among older adults. These findings highlight the gap between evidence and real-world implementation and suggest the need for strategies such as simplified application protocols, enhanced user education, and material improvements to enhance uptake.

Despite relatively high uptake, only 20.1% of participants reported being “very satisfied,” and just 22.6% perceived the interventions as highly effective in relieving insomnia symptoms. This pattern suggests that while TCM techniques were generally acceptable and positively received in community settings, their perceived benefits or service experience rarely reached a level of strong positive evaluation among most users. Such attenuation in satisfaction intensity may reflect real-world implementation factors. Although TCM techniques are increasingly guided by evidence-based principles (43), variability in practitioner training, treatment fidelity, and service delivery settings may limit the consistency of therapeutic effects. In this study, services were delivered across heterogeneous settings, including private clinics, wellness centers, community health service centers, and home-based care. Moreover, more than 60% of participants reported only occasional use or use once or twice per month, which may be insufficient to achieve optimal or sustained symptom improvement. Given the cross-sectional design, it is not possible to determine whether the relatively low proportion of “very satisfied” responses primarily reflects limitations in implementation quality or misalignment between user expectations and achievable treatment effects. Nevertheless, these findings underscore the importance of strengthening implementation quality and patient education to enhance both satisfaction intensity and perceived effectiveness of community-based TCM services.

Predisposing factors such as occupation and awareness significantly influenced TCM technique utilization. Participants who were former enterprise employees or workers were more likely than farmers to use TCM techniques, possibly reflecting socioeconomic differences, higher health literacy, and greater exposure to health information. In contrast, farmers may be more likely to perceive insomnia symptoms as a “normal” part of aging and therefore pay less attention to seeking treatment, supported by evidence showing lower sleep-related healthcare utilization in rural adults (any insomnia symptoms: adjusted OR = 0.45, *p* < 0.001; insomnia disorder: adjusted OR = 0.41, *p* < 0.001) (24). Awareness of TCM techniques emerged as the strongest determinant, consistent with previous findings (36), underscoring the critical role of health education and targeted outreach in improving uptake, particularly in populations with low awareness.

Among enabling factors, having relatives or friends engaged in TCM-related industries may facilitate utilization by providing social support or enhancing TCM health literacy (38), thereby increasing awareness and perceived accessibility of TCM techniques. However, our study did not empirically test these pathways, underscoring the need for future research to clarify the deeper mechanisms. These results highlight the potential of TCM universities to strengthen community engagement and expand outreach programs, which may serve as effective strategies to increase access and reduce disparities in TCM utilization. In addition, community-level interventions, such as integrating TCM services into primary care and leveraging social networks, could further enhance equitable access.

The need factor, insomnia severity, positively predicted TCM use, indicating that perceived or actual health need remains a powerful motivator for seeking care. This aligns with Andersen’s proposition that need factors are the most immediate cause of health service utilization (31) and is consistent with prior evidence showing that symptom burden drives demand for complementary therapies in older adults (24, 36).

Although the observed associations between TCM awareness, insomnia severity, and TCM utilization may appear conceptually intuitive, empirically demonstrating these relationships in community-dwelling older adults remains important. Such evidence supports the applicability of the ABM in the context of TCM use for insomnia and provides a theory-informed basis for designing targeted community interventions. Quantifying these associations also helps identify modifiable targets, such as awareness and perceived need, that can be addressed through health education and service delivery strategies.

### 4.1 Implications for clinical practice, policy, and research

Collectively, these findings provide exploratory insights that may guide future research and inform the development of culturally appropriate interventions within similar contexts.

Clinically, strengthening health education on insomnia and TCM techniques may help improve appropriate uptake and adherence, particularly among older adults with limited awareness. Educational efforts should emphasize realistic expectations, recommended treatment frequency, and potential benefits and limitations of different TCM techniques. In addition, simplifying application protocols and improving materials for techniques such as auricular acupressure may enhance acceptability and sustained use in community settings.

At the policy level, integrating selected TCM services into primary care and community health centers may improve accessibility and continuity of care for older adults with insomnia symptoms. Such integration could help standardize service delivery and reduce variability across practice settings. Importantly, local TCM universities may play a key role in supporting community-based implementation through practitioner training, continuing education, and outreach programs. By leveraging academic expertise and institutional resources, TCM universities could help strengthen practitioner competency, promote evidence-informed practice, and enhance the overall quality and credibility of community TCM services.

Future research should move beyond utilization patterns to examine implementation quality, treatment fidelity, and optimal treatment frequency in real-world settings. Longitudinal and interventional studies are needed to clarify causal relationships between TCM use and insomnia outcomes and to assess whether improvements in practitioner training and service standardization translate into higher satisfaction intensity and perceived effectiveness among older adults.

### 4.2 Limitations and future research

This study has several limitations. First, its cross-sectional design cannot establish causal inferences. Future longitudinal or interventional studies are needed to validate these causal relationships and assess the real-world effectiveness of TCM techniques. Second, TCM use, satisfaction, and perceived effectiveness were self-reported and may be affected by recall and social desirability biases. Although TCM use was assessed specifically for insomnia relief, concurrent use for other coexisting health conditions common in older adults could not be fully distinguished, which may have influenced satisfaction and perceived effectiveness. Future studies may benefit from incorporating more detailed, indication-specific assessments of TCM use to better isolate insomnia-related effects. Third, we did not assess participants’ chronic disease comorbidities and psychological symptoms. The absence of these variables may have led to residual confounding, influencing both insomnia symptoms and treatment-seeking behaviors. Future studies should incorporate comprehensive health and psychological assessments to better account for these potential confounders. In addition, the relatively broad categorization of pre-retirement occupation and place of residence may have limited the ability to capture more nuanced socioeconomic and environmental differences related to TCM utilization. Future studies with larger samples may employ more granular classifications to better capture heterogeneity in TCM use. Finally, participants were recruited from 15 communities in a single city using convenience sampling, which may have resulted in the underrepresentation of frail, homebound, or socially less active older adults and, consequently, may limit the generalizability of the findings. Future research should consider probability-based sampling strategies and multisite designs to improve representativeness and strengthen external validity.

## 5 Conclusion

This exploratory study provides preliminary evidence on the real-world use of TCM techniques for insomnia symptoms among older adults in China. More than half of participants reported using at least one TCM technique, yet only a minority rated the TCM services as “very satisfied” or “highly effective,” suggesting a gap between utilization and expected outcomes. Four associated factors of TCM use were identified under the ABM: pre-retirement occupation and awareness of TCM techniques (predisposing), having relatives or friends working in TCM-related industries (enabling), and insomnia severity (need). These findings may inform the design of culturally sensitive interventions to manage insomnia symptoms among community-dwelling older adults in similar contexts. Future research should continue to assess real-world implementation quality, determinants of perceived effectiveness, and strategies to improve both utilization and user satisfaction.

## Data Availability

The raw data supporting the conclusions of this article will be available from the corresponding author upon reasonable request.
