# Supplementary material for: Utilization, user evaluation, and associated factors of Traditional Chinese Medicine techniques for insomnia symptoms among Chinese community-dwelling older adults: a cross-sectional study
Source: Front Public Health. 2026 Jan 15;13:1746822. doi: 10.3389/fpubh.2025.1746822 (PMC12851957; doi:10.3389/fpubh.2025.1746822)
Supplement: Supplementary file 1 [file Table_1.DOCX]

Supplementary Material

# Questionnaires

**I. General Information**

1. **Sex**: Male / Female
2. **Age** (years): 60–64 / 65–69 / 70–74 / ≥75
3. **Marital status**: Single / Married / Divorced / Widowed
4. **Education level**: Primary school or below / Junior high / High school or technical secondary or vocational school / Junior college / Bachelor / Master or above
5. **Occupation** (before retirement): Civil servant / Public institution staff / Enterprise employee / Worker / Farmer / Self‑employed
6. **Monthly personal income** (Chinese yuan): <2000 / 2000–2999 / 3000–3999 / 4000–4999 / 5000–5999 / ≥6000
7. **Type of medical insurance**: Urban Employee Basic Medical Insurance / Urban and Rural Resident Basic Medical Insurance / None
8. **Place of residence**: Urban / Rural
9. **Are there medical institutions within 5 km of your residence that provide Traditional Chinese Medicine (TCM) techniques?** Yes / No / Not sure
10. **Do you have family members or friends working in TCM‑related industries?** Yes / No

**II. Awareness of TCM Techniques**

1. Do you know the types of TCM techniques?

Completely unaware / Not very aware / Basically aware / Very aware

1. Do you understand the mechanisms of TCM techniques?

Completely unaware / Not very aware / Basically aware / Very aware

1. Do you understand how TCM techniques are performed?

Completely unaware / Not very aware / Basically aware / Very aware

1. Are you aware that TCM techniques are safe and reliable?

Completely unaware / Not very aware / Basically aware / Very aware

1. Are you aware that TCM techniques can be used to relieve insomnia?

Completely unaware / Not very aware / Basically aware / Very aware

1. Are you aware of relevant TCM policies?

Completely unaware / Not very aware / Basically aware / Very aware

**III. Attitude towards TCM Techniques**

1. Overall, what is your attitude towards TCM techniques?

Very opposed / Not very supportive / Relatively supportive/Very supportive

**IV. Insomnia Severity Index (ISI)**

1. Please rate the current (i.e., last week) **SEVERITY** of your insomnia problem(s).

|  | <30 min | 30-45 min | 45-90 min | 90-120 min | >120 min |
| --- | --- | --- | --- | --- | --- |
|  | None | Mild | Moderate | Severe | Very severe |
| **Difficulty falling asleep** | 0 | 1 | 2 | 3 | 4 |
|  |  |  |  |  |  |
| **Difficulty staying asleep** | 0 | 1 | 2 | 3 | 4 |
|  |  |  |  |  |  |
| **Problem waking up too early** | 0 | 1 | 2 | 3 | 4 |

2. How **SATISFIED /DISATISFIED** are you with your current sleep pattern?

| Very satisfied | Satisfied | Neutral | Dissatisfied | Very dissatisfied |
| --- | --- | --- | --- | --- |
| 0 | 1 | 2 | 3 | 4 |

3. To what extent do you consider your sleep problem to **INTERFERE** with your daily functioning (e.g. daytime fatigue, ability to function at work/daily chores, concentration, memory, mood, etc.).

| Not at all | A little | Somewhat | Much | Very much |
| --- | --- | --- | --- | --- |
| Interfering |  |  |  | Interfering |
| 0 | 1 | 2 | 3 | 4 |

4. How **NOTICEABLE** to others do you think your sleeping problem is in terms of impairing the quality of your life?

| Not at all | A little | Somewhat | Much | Very much |
| --- | --- | --- | --- | --- |
| Noticeable |  |  |  | Noticeable |
| 0 | 1 | 2 | 3 | 4 |

5. How **WORRIED /DISTRESSED** are you about your current sleep problem?

| Not at all | A little | Somewhat | Much | Very much |
| --- | --- | --- | --- | --- |
| 0 | 1 | 2 | 3 | 4 |

**V. Use and Satisfaction with TCM Techniques**

1. Have you ever used TCM techniques to relieve insomnia symptoms in the past 12 months? Yes / No
2. For those **who answer “Yes”,** please answer the following 10 questions:
3. Which techniques have you used? (select all that apply):

Acupuncture / needle embedding / Tuina (therapeutic massage) / Moxibustion / Auricular acupressure (ear seeds) / Other (please specify)

1. Average, how frequently do you use TCM techniques?

Daily / 1-2 times a week / 1-2 times a month / Occasionally (≤ 10 times per year)

1. Where do you usually receive services? (select all that apply):

TCM department of general hospital / TCM hospital / Community health service center / Private clinic / Wellness center / Home / Other (please specify)

1. Overall, how satisfied are you with the TCM technology services you received?

Very satisfied / Satisfied / Average / Dissatisfied / Very dissatisfied

1. How satisfied are you with the professionalism of the TCM technology services you received?

Very satisfied / Satisfied / Average / Dissatisfied / Very dissatisfied

1. How satisfied are you with the cost of the TCM technology services you received?

Very satisfied / Satisfied / Average / Dissatisfied / Very dissatisfied

1. How satisfied are you with the convenience of the TCM technology services you received?

Very satisfied / Satisfied / Average / Dissatisfied / Very dissatisfied

1. How satisfied are you with the safety of the TCM technology services you received?

Very satisfied / Satisfied / Average / Dissatisfied / Very dissatisfied

1. How satisfied are you with the privacy protection measures of the TCM technology services you received?

Very satisfied / Satisfied / Average / Dissatisfied / Very dissatisfied

1. How satisfied are you with the effectiveness of the TCM technology services you received in relieving your insomnia symptoms?

Very satisfied / Satisfied / Average / Dissatisfied / Very dissatisfied

# Supplementary Table S1. Multicollinearity Diagnostics for Candidate Independent Variables

| Variables | Collinearity Statistics | |
| --- | --- | --- |
|  | Tolerance | VIF |
| **Education level** |  |  |
| Primary school or below | Reference |  |
| Junior high school | 0.666 | 1.501 |
| High school/Technical secondary/Vocational school | 0.482 | 2.076 |
| Junior college or above | 0.572 | 1.748 |
| **Occupation before retirement** |  |  |
| Farmers | Reference |  |
| Civil servants/Public institution staff | 0.334 | 2.995 |
| Enterprise employees/Workers | 0.459 | 2.178 |
| Self‑employed | 0.741 | 1.349 |
| **Type of medical insurance** |  |  |
| Urban Employee Basic Medical Insurance | Reference |  |
| Urban–Rural Resident Basic Medical Insurance | 0.394 | 2.541 |
| No insurance | 0.646 | 1.547 |
| **Place of residence** |  |  |
| Urban areas | Reference |  |
| Rural areas | 0.775 | 1.290 |
| **Relatives/friends working in TCM‑related industries** |  |  |
| Yes | Reference |  |
| No | 0.956 | 1.046 |
| **Awareness of TCM techniques** |  |  |
| Low | Reference |  |
| Medium | 0.742 | 1.348 |
| High | 0.694 | 1.440 |
| **Insomnia severity** |  |  |
| Mild | Reference |  |
| Moderate | 0.404 | 2.477 |
| Severe | 0.384 | 2.604 |
| TCM: Traditional Chinese Medicine. | | |

# Supplementary Table S2. Statistical Details of Variable Removal in the Backward Stepwise Logistic Regression Model

|  |  |  | Score | df | p |
| --- | --- | --- | --- | --- | --- |
| Step 2^a^ | Variables | **Education level** | 1.179 | 3 | 0.758 |
|  |  | Primary school or below | Reference |  |  |
|  |  | Junior high school | 0.403 | 1 | 0.526 |
|  |  | High school/Technical secondary/Vocational school | 0.126 | 1 | 0.722 |
|  |  | Junior college or above | 0.595 | 1 | 0.440 |
|  | Overall Statistics | | 1.179 | 3 | 0.758 |
| Step 3^b^ | Variables | **Education level** | 0.848 | 3 | 0.838 |
|  |  | Primary school or below | Reference |  |  |
|  |  | Junior high school | 0.548 | 1 | 0.459 |
|  |  | High school/Technical secondary/Vocational school | 0.054 | 1 | 0.816 |
|  |  | Junior college or above | 0.373 | 1 | 0.541 |
|  |  | **Type of medical insurance** | 1.236 | 2 | 0.539 |
|  |  | Urban Employee Basic Medical Insurance | Reference |  |  |
|  |  | Urban–Rural Resident Basic Medical Insurance | 0.873 | 1 | 0.350 |
|  |  | No insurance | 0.007 | 1 | 0.935 |
|  | Overall Statistics | | 2.423 | 5 | 0.788 |
| Step 4^c^ | Variables | **Education level** | 1.043 | 3 | 0.791 |
|  |  | Primary school or below | Reference |  |  |
|  |  | Junior high school | 0.728 | 1 | 0.394 |
|  |  | High school/Technical secondary/Vocational school | 0.078 | 1 | 0.780 |
|  |  | Junior college or above | 0.419 | 1 | 0.518 |
|  |  | **Type of medical insurance** | 0.617 | 2 | 0.734 |
|  |  | Urban Employee Basic Medical Insurance | Reference |  |  |
|  |  | Urban–Rural Resident Basic Medical Insurance | 0.374 | 1 | 0.541 |
|  |  | No insurance | 0.021 | 1 | 0.885 |
|  |  | **Place of residence** |  |  |  |
|  |  | Urban areas | Reference |  |  |
|  |  | Rural areas | 0.760 | 1 | 0.383 |
|  | Overall Statistics | | 3.166 | 6 | 0.788 |
| ^a^ Variable(s) removed on step 2: Education level  ^b^ Variable(s) removed on step 3: Type of medical insurance  ^c^ Variable(s) removed on step 4: Place of residence | | | | | |

**
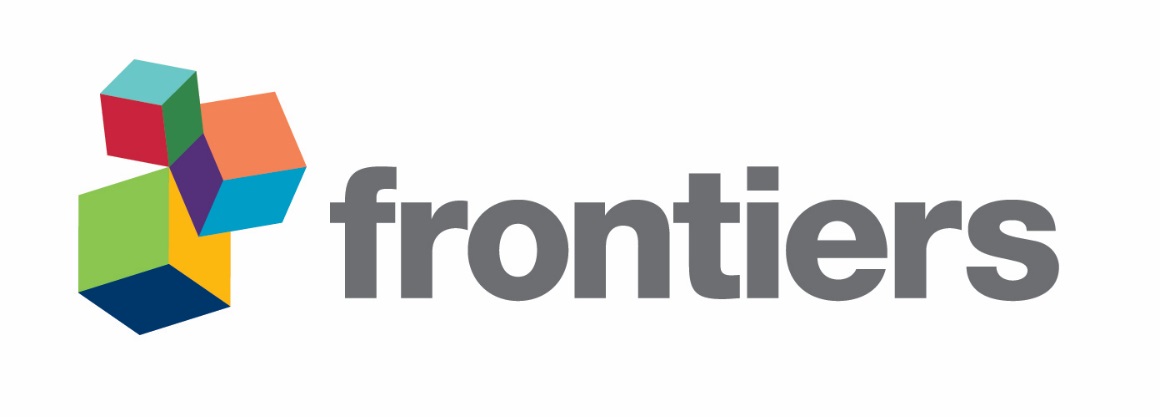
**
